# Adverse events associated with Implantable Collamer Lens: insights from the FDA MAUDE database

**DOI:** 10.3389/fmed.2025.1613060

**Published:** 2025-07-21

**Authors:** Qing Chen, Haijing Yan, Letong Chen, Limei Wang, Qinxiang Zheng, Yueping Ren

**Affiliations:** National Clinical Research Center for Ocular Diseases, Eye Hospital, Wenzhou Medical University, Wenzhou, China

**Keywords:** FDA MAUDE database, adverse events, toric Implantable Collamer Lens, retrospective analysis, spherical Implantable Collamer Lens

## Abstract

**Purpose:**

Implantable Collamer Lens (ICL) implantation is rising globally, yet real-world studies on adverse events (AEs) remain scarce. This study analyses ICL-related complications using the MAUDE database.

**Methods:**

Data on ICL-related AEs were extracted from the MAUDE database. Cases were categorized into spherical and toric ICLs based on lens diopter. Descriptive statistics summarized device-related issues and complications, and Cramér’s V assessed their associations. Management strategies and resolution rates for common complications were also evaluated.

**Results:**

Among 25,001 ICL-related AEs, 43.8% involved spherical ICLs and 56.2% toric ICLs. Common device-related issues included “Shape and/or size problems” (nearly half of annual AEs) and “Off-label use” (around 20%, rising since 2019 and stabilizing). “Operation and control issues” declined steadily since 2019, stabilizing below 3%. Spherical ICL cases with “Activation, positioning, or separation problems” dropped below 1% post-2019, while toric cases remained at 6.9–9.0%. The top three complications—"Vision issues,” “Intraocular pressure issues” and “Lens-related issues”—were most frequent, with “No patient impact” in most cases. Moderate correlations were found between complications and ICL-related AEs (*p* = 9.999 × 10^−5^, Cramér’s V: 0.47 for spherical, 0.45 for toric). Management strategies, including lens exchange and lens extraction followed by surgery, demonstrated high resolution rates.

**Conclusion:**

While ICL implantation is generally safe, concerns about inappropriate sizing and off-label use persist. This study suggests that improving lens sizing accuracy and adhering to guidelines may reduce AEs.

## Introduction

Phakic intraocular lens (pIOL) is a promising alternative for correcting refractive error, especially for patients with moderate-to-high ametropia whose corneas are not suitable or contraindicated for corneal refractive procedures (1). This category of refractive surgery has been rapidly growing both in the United States and worldwide. The Implantable Collamer Lens (ICL) (STAAR Surgical, Nidau, Switzerland), the predominantly used posterior chamber pIOL, has experienced significant usage, with over 3 million ICLs implanted worldwide to date, compared to 1 million in 2019 and 2 million in 2022 (2, 3), underscoring its increasing acceptance and widespread use.

The ICL implantation is an effective procedure to treat myopia and myopia with astigmatism (4), but its complications can be more serious than keratorefractive surgery. Complications and long-term safety concerns include endothelial cell loss, cataract formation, secondary glaucoma (e.g., pupillary block and pigment dispersion), iris atrophy (e.g., pupil ovalization), traumatic dislocation, uveitis, and endophthalmitis (5, 6). A 10-year follow-up study on postoperative outcomes of ICL implantation reported an average endothelial cell loss of 5.3% at the 10-year mark and an anterior subcapsular cataract formation incidence of 10.5% during the 5 to 10-year follow-up period, with no vision-threatening complications noted throughout the follow-up (7). Additionally, a review on ICL implantation indicated that the incidence of intraocular pressure (IOP) complications ranged from 0 to 19.9%, the endothelial cell loss rate varied between 0.1–22.0%, and the rate of cataract formation ranging 0–3.85% (1, 8). However, the existing data on related complications show inconsistent results, with studies having single-center designs, small sample sizes, and potential selection bias. Moreover, intraoperative and postoperative complications are not only associated with inherent issues in ICL design but may also be influenced by improper ICL sizing and insufficient surgeon expertise. Nevertheless, comprehensive data on device-related issues and associated ICL complications remain limited.

The United States Food and Drug Administration’s (FDA) Manufacturer and User Facility Device Experience (MAUDE) database is a critical online repository that contains millions of medical device reports (MDRs) submitted annually by mandatory reporters, such as manufacturers, importers, and device user facilities. Additionally, a major advantage of the MAUDE database is its inclusion of reports from ophthalmologists with diverse backgrounds and practice settings, making the data more representative. Since its public availability in 1999, the MAUDE database has served as a vital resource for monitoring the performance of medical devices and identifying adverse events (AEs) associated with them (9). It provides real-world data that complements pre-market clinical trials, offering insights into device-related complications, malfunctions, and safety concerns that may not be evident during initial testing. This database has been widely utilized in research to investigate AEs associated with various medical technologies, including assistive technologies in ophthalmic surgeries (10), ophthalmic injectable drugs (11), and human implants (12–14). By analyzing reports of suspected device-associated deaths, serious injuries, and malfunctions, the MAUDE database helps patients, healthcare providers, and regulators make more informed decisions, ultimately enhancing patient safety and driving improvements in device design and clinical practice. Its role in facilitating post-market surveillance and supporting evidence-based medical decision-making underscores its importance as a cornerstone of medical device safety and regulatory oversight.

## Materials and methods

As the data is publicly accessible, de-identified, and does not involve new patient data collection, approval of the institutional review board (IRB) and ethical committee oversight were not required.

### Study population

We extracted all pIOL-related AEs reported between 2015 and 2023 (Supplementary Data File 1, 2), with the detailed screening process depicted in Supplementary Figure 1. To ensure the specificity of our analysis, only adverse event reports explicitly linked to the ICL models were included. Demographic and clinical data—including lens diopter, patient age, gender, laterality of the surgical eye, event date, and reporter occupation—were retrieved and analyzed. Duplicate reports were removed based on identical values in fields, such as patient demographics, event details, and report numbers. Reports from publications, entries with missing or indeterminable lens diopter values, and those with positive diopter values were excluded. The final dataset was categorized into spherical ICL and toric ICL groups based on the lens diopter for further analysis. The extracted raw data contained 123 device-related issues and 139 patient-related issues. Through consolidation of semantically similar items, these were systematically refined into 9 device issues (Activation, positioning or separation problem, Patient-Device interaction issues, etc.) and 8 patient issues (Vision issues, IOP issue, etc.) (The details were presented in Supplementary Data File 3, 4). The top three patient-related complications, “vision issues,” “intraocular pressure issues,” and “lens-related issues,” were assessed for management and prognostic outcomes.

### Statistical analysis

Summary statistics were computed for all variables, including patient demographics, device issues, patient issues, subsequent interventions, and reporter occupation. Chi-squared tests assessed differences between groups, with standardized residuals exceeding ±1.96 deemed significant at the 5% levels (15).

Annual trends in complication reporting were evaluated by classifying complications by reporting year and determining their proportions. A secondary analysis was conducted to assess the correlations between the top three patient complications and device-related issues, along with their management strategies and outcomes.

To address low expected cell counts in contingency tables, we used Monte Carlo simulations with 10,000 replicates to estimate *p* values, ensuring robustness for sparse or small-sample data (16).

Cramér’s V, calculated using the CramerV function from the DescTools R package, quantified the strength of associations between categorical variables, with values interpreted as follows: 0 to 0.1 denotes little-to-no or very weak association; 0.1 to 0.3 indicates a weak association; 0.3 to 0.5 represents a moderate association; and above 0.5 signifies a strong association (17).

We selected Cramér’s V over the Chi-square test for assessing associations in large R × C contingency tables because the Chi-square test becomes less reliable when dealing with high-dimensional or sparse data, where many cells have low expected counts (18). Additionally, the Chi-square test is highly sensitive to sample size and does not provide a direct measure of association strength. In contrast, Cramér’s V offers a normalized measure ranging from 0 to 1, which is less affected by sample size and allows for more intuitive interpretation of the strength of relationships across complex categorical data structures (19).

All analyses were performed with R software (version 4.3.1). *p* < 0.05 was considered statistically significant.

## Results

### Demographic and baseline clinical factors of included patients

The MAUDE database recorded 27,039 patients who underwent pIOL implantation between 2015 and 2023, of whom 25,001 met the study criteria. Of these, 10,953 patients (43.8%) received spherical ICL implants, while 14,048 patients (56.2%) received toric ICL implants (Supplementary Data 5, 6). Demographics and baseline clinical factors for these patients are summarized in Table 1. Most patients in both groups were between 21 and 35 years old. The left-to-right eye ratio was approximately 1:1, with the majority of reports submitted by physicians. Chi-square tests were used to assess the statistical significance of the differences between the two groups, and highly significant *p* (<2.2 × 10^−16^) was observed across all variables. Supplementary Table 1 provides the residual analysis for each category, highlighting the contributions of specific subgroups to the overall differences. Standardized residuals revealed significant deviations, particularly for gender (e.g., females and males, |residual| > 12), certain age cohorts, and surgical years (e.g., 2015 and 2023, |residual| > 15), indicating notable subgroup-level disparities in the distribution between the two groups.

**Table 1:** Demographic and baseline clinical factors of included patients (spherical ICL and toric ICL).

Variable	Spherical ICL (*n* = 10,953)	Toric ICL (*n* = 14,048)	*P* value
Patient age at surgery (yrs.)			
<21	787	1,563	<2.2e-16
21–25	2,178	3,295	
26–30	2,487	3,129	
31–35	2058	2,308	
36–40	1,285	1,384	
41–45	722	726	
>45	446	411	
Missing age	990	1,232	
Year			
2015	716	365	<2.2e-16
2016	951	503	
2017	906	690	
2018	803	811	
2019	996	1,188	
2020	1,277	1708	
2021	1922	2,729	
2022	1,663	2,677	
2023	1719	3,377	
Reporter occupation			
Administrator/supervisor	4	4	<2.2e-16
Health professional	10	0	
Non-healthcare professional	13	12	
Nurse	81	3	
Other	479	56	
Other health care professional	250	296	
Paramedic	0	1	
Patient	70	3	
Pharmacist	1	0	
Physical therapist	0	2	
Physician	7,532	10,478	
Physician assistant	9	13	
Not reported	2,504	3,180	
Eye laterality			
Left	4,727	6,482	<2.2e-16
Right	5,154	6,935	
Not reported	1,072	631	

ICL, Implantable Collamer Lens.

### Device issues

For device issues (Supplementary Table 2), the most frequently reported issues for both spherical ICL and toric ICL groups were “Shape and/or size problem” and “Off-label use,” with 66.8 and 21.4% for spherical ICL, and 69.1 and 21.8% for toric ICL, respectively. For “Optical problem,” 915 cases (8.4%) were reported in the spherical ICL group, compared to 1,498 cases (10.7%) in the toric ICL group. Notably, “Activation, positioning, or separation problem” was reported in 183 cases (1.7%) for spherical ICL and 1,644 cases (11.7%) for toric ICL.

To enhance clarity regarding device issue classifications, Tables 2, 3 summarizes the specific subcategories included under major MAUDE device issue codes, such as “activation, positioning or separation problem,” “operation and control issues,” and others. These subcategories were extracted directly from the MAUDE coding and provide insight into the specific types of complications involved.

**Table 2:** Distribution of spherical ICL device issues by category.

Device issues	Counts
Activation, positioning or separation problem	183
Activation failure	9
Activation, positioning or separation problem	3
Device dislodged or dislocated	112
Positioning failure	42
Others	20
Patient-device interaction issues	146
Patient device interaction problem	12
Patient-device incompatibility	134
Off-label use	2,340
Operation and control Issues	846
Device operates differently than expected	569
Improper or incorrect procedure or method	42
Unintended movement	241
Others	2
Optical issues	915
Misfocusing	735
Optical distortion	2
Optical problem	229
Shape and/or size issues	7,322
Inadequacy of device shape and/or size	7,322
No AEs	1,164
No code available	1,699
Other	308

**Table 3:** Distribution of toric ICL device issues by category.

Complication	Counts
Activation, positioning or separation problem	1,644
Activation failure	3
Device dislodged or dislocated	1,619
Ejection problem	9
Positioning failure	7
Others	8
Patient-device interaction issues	188
Patient device interaction problem	16
Patient-device incompatibility	172
Off-label use	3,058
Operation and control Issues	647
Device operates differently than expected	369
Improper or incorrect procedure or method	65
Unintended movement	225
Others	2
Optical issues	1,498
Misfocusing	1,296
Optical decentration	3
Optical problem	255
Shape and/or size issues	9,710
Inadequacy of device shape and/or size	9,710
No AEs	1,166
No code available	1,175
Other	141

**Figure 1:**
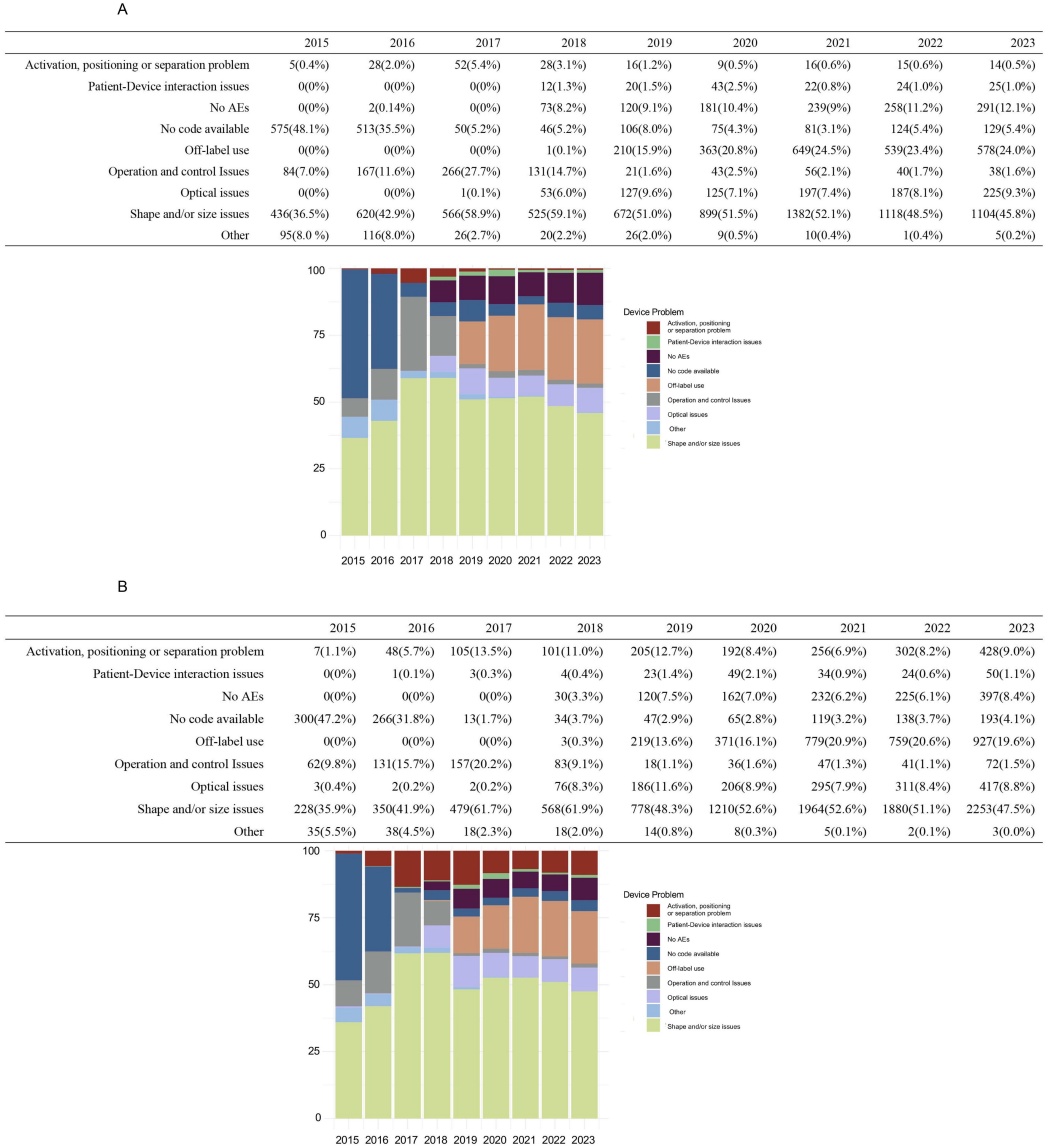
Annual distribution of device-related issues for spherical ICL and toric ICL groups 2015–2023. **(A)** The annual distribution of device-related problems for the spherical ICL group from 2015 to 2023, categorized as activation, positioning, or separation problems, patient–device interaction issues, no adverse events (AEs), no code available, off-label use, operation and control problems, optical issues, shape and/or size issues, and other issues. **(B)** The corresponding distribution for toric ICL-related device issues over the same period. The *y*-axis indicates the percentage of cases, while the *x*-axis represents the years.

Figure 1 illustrates the annual trends in device-related issue reporting. Reports without specific codes accounted for a significant proportion of events before 2017, declining sharply after that. Consequently, subsequent analyses focused on post-2016 data to minimize potential bias. “Shape and/or size problem” consistently accounted for approximately half the reported device-related AEs annually. Reports of “Off-label use” were relatively low in 2017 and 2018 but surged in 2019, stabilizing around 20% after that. Conversely, “Operation and control issues” showed a decline from 2019, stabilizing below 3% in subsequent years. Significant differences in the reporting of “Activation, positioning, or separation problems” were observed between the two groups, with spherical ICL cases declining below 1% post-2019, while toric ICL cases remained between 6.9 and 9.0%.

### Patient issues

**Figure 2:**
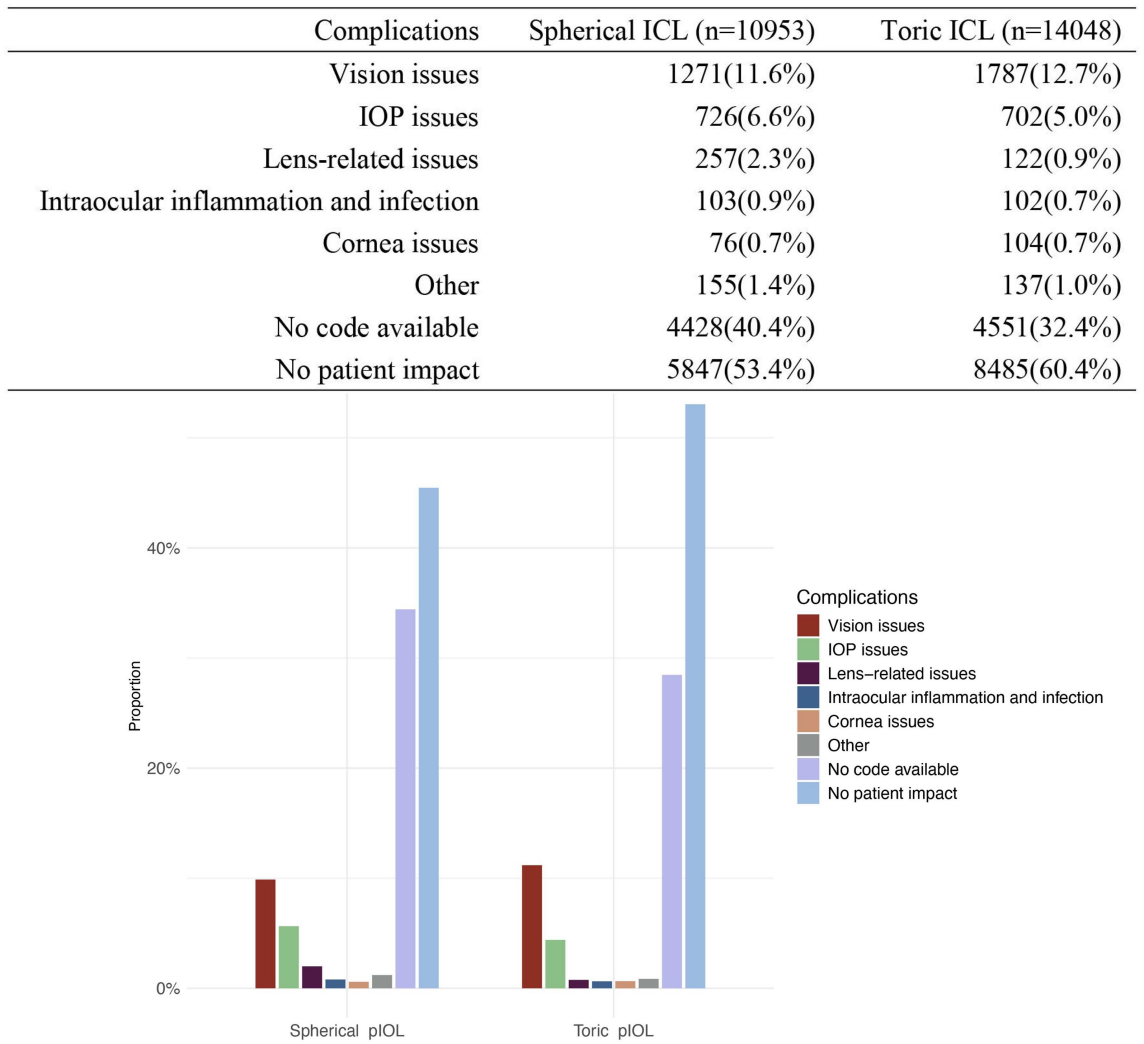
Distribution of complications for patients with spherical ICL and toric ICL. The figure compares the proportion of complications between patients of spherical ICL and toric ICL groups. Complications are categorized into vision issues, intraocular pressure (IOP) issues, lens-related issues, intraocular inflammation and infection, corneal issues, other complications, no code available, and no patient impact. The percentages for each category are shown for both groups. The *y*-axis represents the proportion of cases, and the *x*-axis distinguishes between the two groups.

Reports indicating “No patient impact” were 5,847 cases (53.4%) in the spherical ICL group and 8,485 cases (60.4%) in the toric ICL group. Additionally, 4,428 spherical ICL and 4,551 toric ICL cases were classified as “No code available.” The most common issues were “Vision issues” (spherical ICL: 11.6%; toric ICL: 12.7%), “IOP issues” (spherical ICL: 6.6%; toric ICL: 5.0%), and “Lens-related issues” (spherical ICL: 2.3%; toric ICL: 0.9%). To enhance clarity regarding patient issue classifications, Tables 4, 5 summarizes the specific subcategories included under major MAUDE patient issue codes, such as “Vision issues,” “IOP issues,” and “Lens-related issues.” These subcategories were extracted directly from the MAUDE database and provide insight into the nature of patient-related complications. Figure 2 presents a comprehensive overview of patient-related issues.

**Table 4:** Distribution of spherical ICL patient issues by category.

Complications	Counts
Vision issues	1,271
Blurred vision	908
Fatigue	2
Halo	310
Loss of vision	38
Visual disturbances	328
Visual impairment	18
IOP issues	726
Glaucoma	21
Intraocular pressure increased	618
Intraocular pressure, delayed, uncontrolled	19
Pupillary block	73
Others	169
Lens-related issues	257
Cataract, induced	223
Cataract	62
Others	4
Intraocular inflammation and infection	103
Endophthalmitis	36
Hypopyon	4
Inflammation	54
Iritis	2
Uveitis	10
Others	4
Cornea issues	76
Corneal decompensation	9
Corneal edema	67
Others	7
No code available	4,428
No patient impact	5,847
Others	155

**Table 5:** Distribution of toric ICL patient issues by category.

Complications	Counts
Vision issues	1787
Blurred vision	1,456
Fatigue	3
Halo	303
Loss of vision	20
Visual disturbances	324
Visual impairment	16
IOP issues	702
Glaucoma	20
Intraocular pressure decreased	1
Intraocular pressure increased	626
Intraocular pressure, delayed, uncontrolled	5
Pupillary block	70
Others	131
Lens-related issues	122
Cataract	113
Cataract, induced	12
Others	3
Intraocular inflammation and infection	102
Endophthalmitis	14
Hypopyon	3
Inflammation	61
Iritis	20
Uveitis	18
Others	4
Cornea issues	104
Corneal decompensation	15
Corneal edema	89
Others	6
No code available	4,551
No patient impact	8,485
Others	137

### Secondary analyses

**Figure 3:**
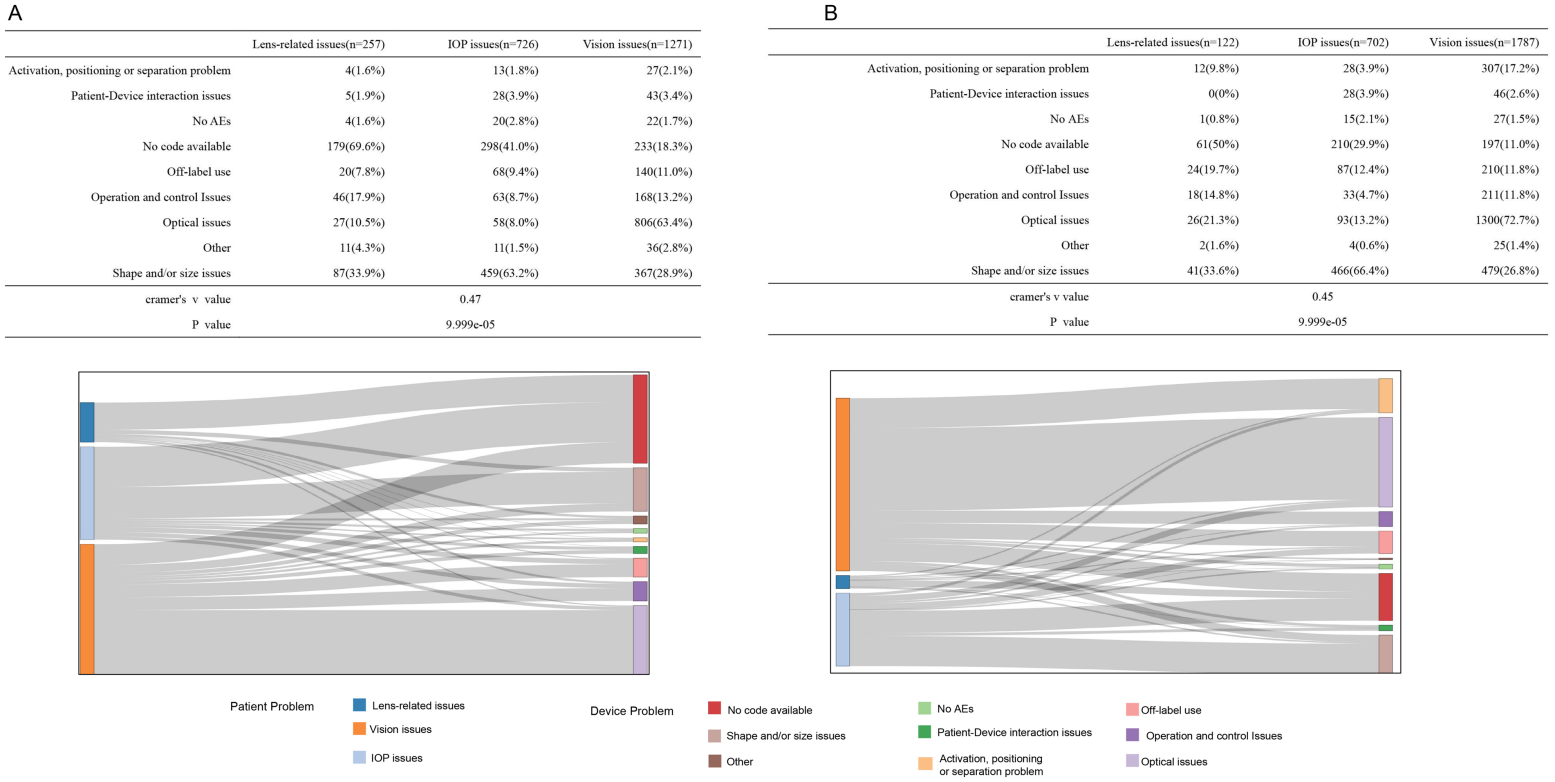
Association of the top-three patient issues with device-related problems for the spherical ICL **(A)** and toric ICL **(B)** groups. **(A)** The association between the top three patient-reported issues (lens-related, IOP, and vision issues) and various device problems for the spherical ICL group, with corresponding percentages shown in the table. The Sankey diagram illustrates the flow of associations between patient issues and device problems, including no code available, shape/size issues, optical issues, and other categories. **(B)** The same analysis is for toric ICL group patients. Cramér’s *V* values are provided to quantify the strength of the association between patient issues and device problems for the spherical ICL group (*V* = 0.47) and the toric ICL group (*V* = 0.45).

Figure 3 illustrates the association between the three types of patient problems and nine device issues. The tables and Sankey diagrams indicate that, for spherical ICL-related complications, lens-related issues were predominantly attributed to “Shape and/or size issues” (33.9%), “Operation and control issues” (17.9%), and “Optical issues” (10.5%). IOP issues were mainly linked to “Shape and/or size issues” (63.2%), “Off-label use” (9.4%), and “Operation and control issues” (8.7%). Vision issues were primarily associated with “Optical issues” (63.4%), “Shape and/or size issues” (28.9%), and “Operation and control issues” (13.2%). For toric ICL-related complications, lens-related issues are linked to “Shape and/or size issues” (33.6%), “Operation and control issues” (14.8%), and “Off-label use” (19.7%). IOP issues were strongly connected to “Shape and/or size issues” (66.4%), “Optical issues” (13.2%), and “Off-label use” (12.4%). Vision issues were largely tied to “Optical issues” (72.7%), “Shape and/or size issues” (26.8%), and “Activation, positioning, or separation problems” (17.2%). The Monte Carlo analysis (10,000 replicates) showed significant associations (*p* = 9.999 × 10^−5^), with a moderate correlation between patient complications and device malfunctions (Cramér’s *V*: 0.47 for spherical ICL; 0.45 for toric ICL).

**Figure 4:**
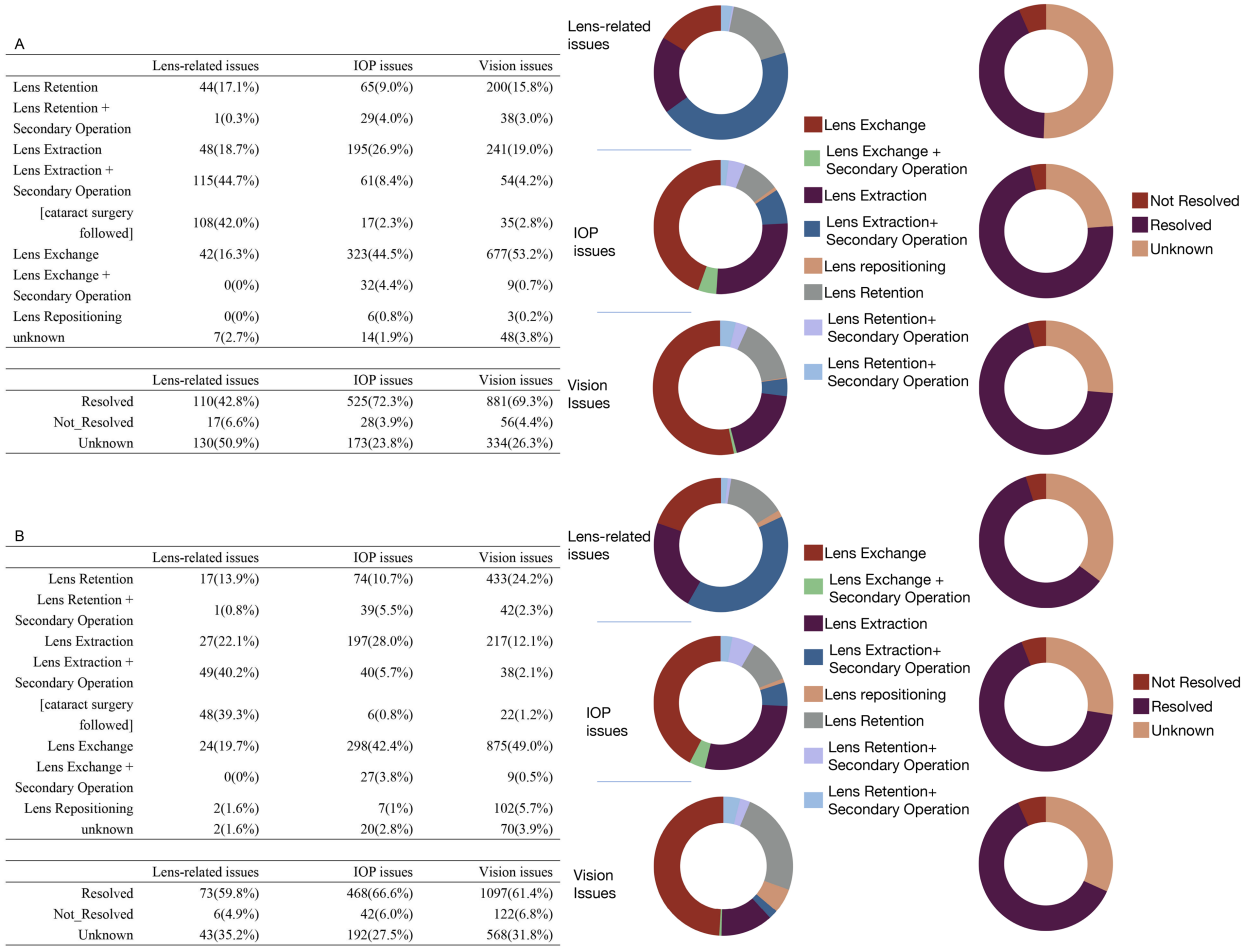
Treatment methods and outcomes for the top three patient issues in the spherical ICL **(A)** and toric ICL **(B)** groups. **(A)** The treatment approaches and outcomes for patients of the spherical ICL group with the top three issues: lens-related, intraocular pressure (IOP), and vision issues. The upper table details the specific treatments applied, while the lower table shows the resolution status (resolved, not resolved, or unknown) for each issue. The two sets of donut charts to the right visually represent the distribution of treatment methods (left) and outcomes (right). **(B)** Similar data for toric ICL group patients follow the same structure as **(A)**, with tables for treatment methods and outcomes accompanied by corresponding donut charts on the right.

Figure 4 summarizes the primary intervention methods and outcomes for vision, IOP, and lens-related issues. For vision and IOP issues, lens exchange—defined as the replacement of the original ICL with another ICL of a different specification—was the most commonly utilized intervention, representing the largest proportion in these categories. In contrast, for lens-related issues, the most frequently performed intervention was lens extraction followed by a secondary operation, the majority of which involved subsequent cataract surgery, accounting for nearly half of all such cases. In terms of outcomes, the top three complications—vision issues, IOP issues, and lens-related issues—demonstrated high-resolution rates. Vision and IOP issues had favorable prognoses, with over 60% of cases resolved in both spherical ICL and toric ICL groups. Although lens-related problems also exhibited notable resolution rates, a substantial proportion of outcomes remained undetermined, underscoring the need for improved follow-up and documentation.

## Discussion

Our analysis of MAUDE data from 2015 to 2023 identified “shape and/or size issues” as the most frequently reported device-related problem, underscoring the importance of accurate ICL sizing in clinical outcomes. This finding aligns with prior literature suggesting that improper sizing is a key contributor to several complications. Oversized lenses may cause excessive vaulting, which can increase intraocular pressure through mechanisms such as angle-closure, pigment dispersion, or pupillary block (20). In contrast, undersized lenses can result in low vaults, lens rotation, and early cataract formation (21). Although current sizing strategies often rely on white-to-white measurements and anterior chamber depth, these approaches have inherent limitations (22). Our findings emphasize the need for continued refinement of sizing techniques to minimize vault-related complications and improve patient safety.

“Off-label use” is the second most common device issue, with a relatively low incidence in 2017 and 2018. However, it began to rise in 2019 and has since maintained a significant proportion. The trend in “Operation and control issues” exhibits an inverse correlation with “Off-label use,” showing elevated percentages in 2017 and 2018, followed by a significant decrease commencing in 2019, remaining below 3% in subsequent years. Given the introduction of the V5 model with a larger optical diameter in 2016 (23), it is plausible that users initially encountered a learning curve with new or updated products, resulting in a more cautious adherence to labeling guidelines. As experience increases, people may develop confidence in their methods. Nonetheless, it is crucial to emphasize that “Off-label use” represents a substantial portion of the top three complications in patients, ranging from 5.2–15% in both spherical ICL and toric ICL groups. This serves as an important reminder for practitioners to rigorously follow guidelines and labeling requirements to enhance safety and improve patient outcomes.

In the MAUDE database, reports of “No patient impact” accounted for the most patient-related AEs associated with ICL. The top three patient complications in both spherical ICL and toric ICL groups were “Vision issues,” “IOP issues,” and “Lens-related issues.” These findings align with previous studies indicating that complications after ICL implantation often involve increased IOP and corneal, lens, or retinal complications (24, 25). It is noteworthy that “Vision issues” was the most frequent patient-reported complication in MAUDE, despite earlier research suggesting comparable or better outcomes with ICL compared to corneal laser refractive surgery. This emphasizes the efficacy and reliability of ICL for refractive correction, which remains a significant issue. The FDA has provided specific standards to assess the effectiveness and predictability of outcomes following ICL implantation (26). Additionally, visual impairments may correlate with myopic regression and the formation of lens opacities over time, along with a deterioration in the optical characteristics of the lens post-ICL implantation (7).

Using Cramér’s *V*, we identified a moderate association between device issues and the three primary patient complications, suggesting that device-related factors may influence the incidence of patient complications. Notably, “Vision issues” were most closely associated with “Optical issues” in both groups. A 10-year retrospective follow-up study on ICL implantation for the correction of myopia and myopic astigmatism indicated that the safety and efficacy of toric ICL were comparable to those of non-toric ICL (7). Interestingly, in the toric ICL group, nearly 20% of “Vision issues” were associated with “Activation, positioning, or separation problems,” compared to only 2.1% in the spherical ICL group. The annual complication reports reveal that “Activation, positioning or separation problems” in the spherical ICL group decreased to below 1% post-2019, whereas incidents in the toric ICL group persisted between 6.9 and 12.7%. These results underscore the necessity for increased vigilance regarding positioning when utilizing toric lenses. IOP-related complications were primarily associated with “Shape and/or size issues.” As previously discussed, an oversized lens may result in a high vault, subsequently increasing IOP and leading to related complications (20).

Lens-related issues were strongly associated with “Shape and/or size issues.” Cataract formation in ICL patients may result from aging, lens trauma, or inadequate aqueous humor circulation (25). Aging is an unavoidable physiological change, and improper lens sizing can cause substantial contact between the ICL and natural lens, leading to cataract formation. The aquaport feature in newer ICL designs promotes aqueous flow, reducing the need for peripheral iridectomy and cataract risk (1). Surgeon experience, careful irrigation, and intraoperative technique also influence the lens opacity, as noted by Montes-Mico et al. (4). This may explain why “Operation and control issues” constitute over 10% of lens-related issues reports in both groups.

The statistical findings for the subsequent management of the three main complications indicate that the major treatment strategies are identical for both the spherical ICL and toric ICL groups. In cases of vision issues, approximately 50% involved lens exchange, which is also used to manage IOP issues. For lens-related issues, the main approach was lens extraction combined with secondary procedures. Following adequate follow-up management, the majority of issues associated with these three primary complications were addressed. João Heitor Marques et al. reported that the most common reasons for pIOL explantation are cataract formation and endothelial cell loss. After the timely removal of pIOL, both vision and endothelial cell density showed sustained improvement (27). A retrospective investigation on double lens extraction for eyes with pIOL corroborated this perspective while also indicating that some vision-threatening problems may occur post-extraction, including retinal detachment and diminished endothelial cell density, the latter being more prevalent (28). Additionally, the incidence of complications and the irreversible damage they may cause often necessitate further surgeries, which can impose economic burdens, psychological distress, and physical harm to patients.

This study has several limitations. Firstly, the lack of a known denominator prevented incidence rate estimation. Secondly, the use of MAUDE procedural term codes and electronic reports introduces inherent limitations, including underreporting—particularly those deemed minor or those effectively managed without further intervention, duplicate submissions, misclassification, and missing data. Importantly, the semantic ambiguity of reported terms—as well as variability in reporting sources (e.g., physicians, patients, or manufacturers)—may compromise data accuracy and consistency. In addition, some terminology was overly broad and lack the granularity needed for precise clinical interpretation. These semantic limitations, inherent to the MAUDE reporting system, may affect the clarity of our analysis and reduce its direct applicability in guiding clinical practice. Misclassification, in particular, could impact comparisons between different lens types. To mitigate this, we excluded cases with missing lens power or obvious duplication, and categorized data based on available lens power information. Furthermore, although we provide statistical summaries of adverse event reports, these data do not represent true incidence or prevalence rates. The MAUDE database lacks denominator information which limit its ability to yield population-level risk estimates. Therefore, caution should be exercised when interpreting the frequency of complications, and comparisons with findings from prospective clinical trials or registry-based studies should be avoided. Furthermore, although the MAUDE database enables large-scale analyses of device-related complications, it lacks global representation and may be affected by selection and reporting biases. These limitations underscore the importance of cautious interpretation. Nonetheless, we believe our findings are valuable in highlighting the real-world diversity of complications associated with ICL and in revealing longitudinal trends in adverse event reporting. Future research could benefit from multi-center collaboration and integration of international datasets to improve generalizability and data reliability.

Although several of the complications identified in our study—such as sizing problems, elevated intraocular pressure, and cataract formation—have been previously documented in clinical studies, the strength of our analysis lies in its use of a large-scale public adverse event database. This study should be interpreted not as a definitive clinical investigation, but rather as a real-world post-market surveillance summary. By analyzing trends and distributions of reported events from diverse sources, our findings contribute to ongoing safety monitoring and highlight key areas where further clinical research and regulatory attention are warranted.

## Conclusion

This study offers a descriptive summary of post-market adverse events associated with ICL reported in the MAUDE database from 2015 to 2023. While the majority of cases indicated no patient impact, reported complications underscore the importance of appropriate lens sizing and adherence to clinical guidelines. This analysis provides valuable insights into safety signals and reporting trends that may inform future research and clinical vigilance.

## Data Availability

The original contributions presented in the study are included in the article/Supplementary material, further inquiries can be directed to the corresponding authors.

## Supplementary material

SUPPLEMENTARY FIGURE 1 Flowchart of the data screening process for device analysis.
