# Supplementary figures and images for: Adverse events associated with Implantable Collamer Lens: insights from the FDA MAUDE database
Source: Front Med (Lausanne). 2025 Jul 21;12:1613060. doi: 10.3389/fmed.2025.1613060 (PMC12319018; doi:10.3389/fmed.2025.1613060)

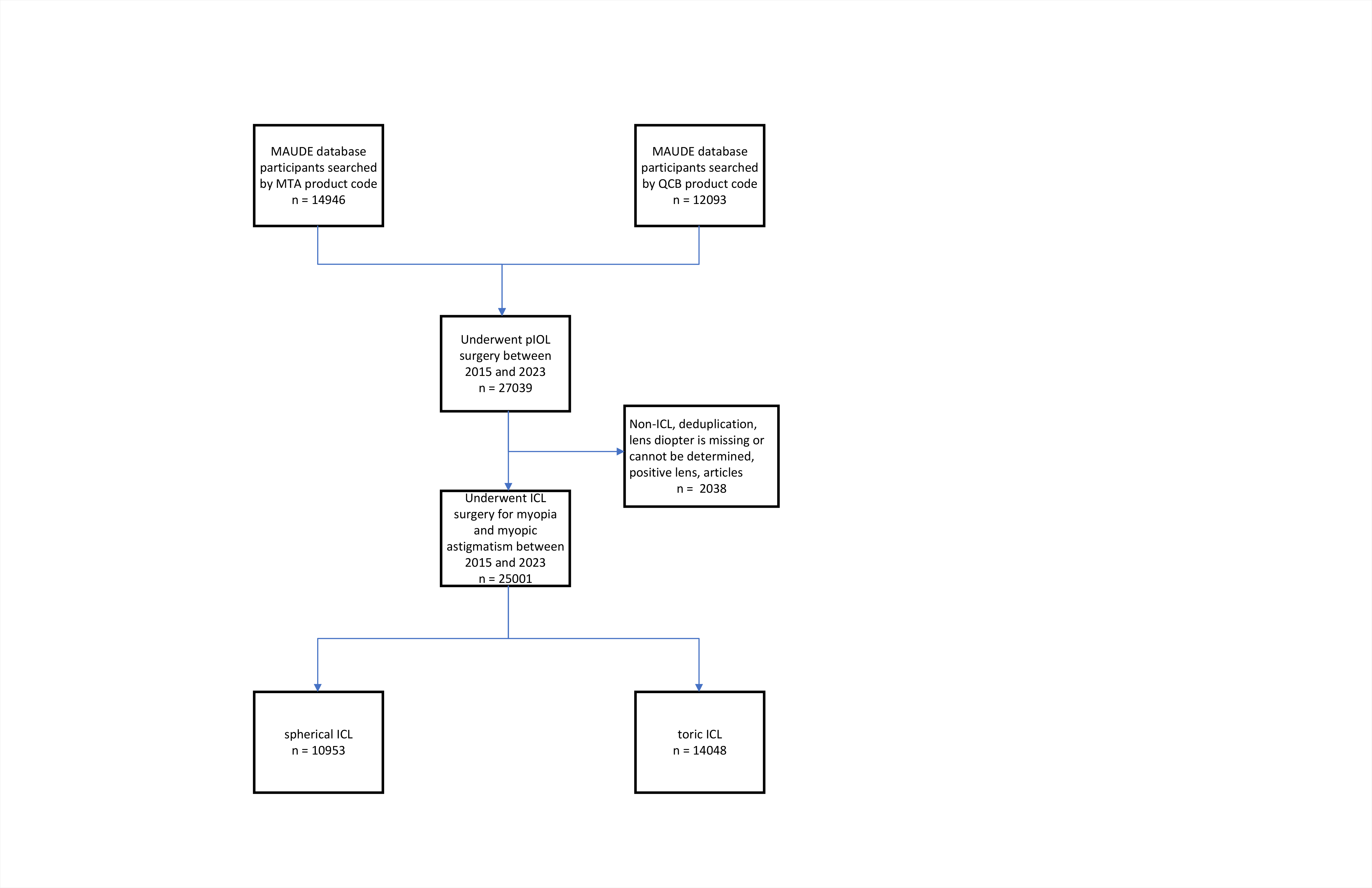

Supplement: SUPPLEMENTARY FIGURE 1 — Flowchart of the data screening process for device analysis. [file Image_1.TIF]
