# Supplementary material for: Adverse events associated with Implantable Collamer Lens: insights from the FDA MAUDE database
Source: Front Med (Lausanne). 2025 Jul 21;12:1613060. doi: 10.3389/fmed.2025.1613060 (PMC12319018; doi:10.3389/fmed.2025.1613060)
Supplement: Supplementary file 2 [file Table_1.DOCX]

**Supplementary Table 1.** Residual Analysis of Demographic Characteristics and Baseline Clinical Factor Between the Spherical pIOL and Toric pIOL Groups

| Patient age at surgery (yrs) | Spherical pIOL | Toric pIOL |
| --- | --- | --- |
| ＜21 | -10.599584 | 10.5995836 |
| 21-25 | -6.7647682 | 6.7647682 |
| 26-30 | 0.8071851 | -0.8071851 |
| 31-35 | 4.8683817 | -4.8683817 |
| 36-40 | 4.7699349 | -4.7699349 |
| 41-45 | 4.7519652 | -4.7519652 |
| ＞45 | 4.9328382 | -4.9328382 |
| Missing age | 0.7957004 | -0.7957004 |
| Gender |  |  |
| Female | -12.75416 | 12.75416 |
| Male | -19.06526 | 19.06526 |
| Not reported | 25.07618 | -25.07618 |
| Year |  |  |
| 2015 | 15.189846 | -15.189846 |
| 2016 | 17.069573 | -17.069573 |
| 2017 | 10.753619 | -10.753619 |
| 2018 | 4.971763 | -4.971763 |
| 2019 | 1.765735 | -1.765735 |
| 2020 | -1.167537 | 1.167537 |
| 2021 | -3.773309 | 3.773309 |
| 2022 | -8.026515 | 8.026515 |
| 2023 | -16.255156 | 16.255156 |
| Reporter occupation |  |  |
| Administrator/Supervisor | 0.7101076 | -0.7101076 |
| Health Professional | 3.5817808 | -3.5817808 |
| Non-Healthcare Professional | 0.8254036 | -0.8254036 |
| Nurse | 9.7355536 | -9.7355536 |
| Other | 21.5452761 | -21.545276 |
| Other Health Care Professional | 0.9400065 | -0.9400065 |
| Paramedic | -0.8830729 | 0.8830729 |
| Patient | 8.9809374 | -8.9809374 |
| Pharmacist | 1.1324547 | -1.1324547 |
| Physical Therapist | -1.2488786 | 1.2488786 |
| Physician | -10.21364 | 10.2136403 |
| Physician Assistant | -0.274687 | 0.274687 |
| Not reported | 0.4489895 | -0.4489895 |
| Eye laterality |  |  |
| Left | -4.728239 | 4.728239 |
| Right | -3.605476 | 3.605476 |
| Not reported | 16.485993 | -16.485993 |

Abbreviations: pIOL: phakic intraocular lens.
