# Supplementary material for: Adverse events associated with Implantable Collamer Lens: insights from the FDA MAUDE database
Source: Front Med (Lausanne). 2025 Jul 21;12:1613060. doi: 10.3389/fmed.2025.1613060 (PMC12319018; doi:10.3389/fmed.2025.1613060)
Supplement: Supplementary file 3 [file Table_2.DOCX]

**Supplementary Table 2.** Device Issues Reported for the Spherical ICL and Toric ICL Groups

| Device Issues | Spherical ICL (n=10953) | Toric ICL  (n=14048) |
| --- | --- | --- |
| Activation, positioning or separation problem | 183(1.7%) | 1644(11.7%) |
| Patient-Device interaction issues | 146(1.3%) | 188(1.3%) |
| No AEs | 1164(10.6%) | 1166(8.3%) |
| No code available | 1699(15.5%) | 1175(8.4%) |
| Off-label use | 2340(21.4%) | 3058(21.8%) |
| Operation and control Issues | 846(7.7%) | 647(4.6%) |
| Optical issues | 915(8.4%) | 1498(10.7%) |
| Shape and/or size issues | 7322(66.8%) | 9710(69.1%) |
| Other | 308(2.8%) | 141(1.0%) |

Abbreviations: pIOL: phakic intraocular lens; AEs: adverse events.
